# Competitive Binding of Bilirubin and Fatty Acid on Serum Albumin Affects Wear of UHMWPE

**DOI:** 10.3390/lubricants8050053

**Published:** 2020-05-10

**Authors:** Spencer Fullam, Jade He, Caroline S. Scholl, Thomas M. Schmid, Markus A. Wimmer

**Affiliations:** 1 Department of Orthopedic Surgery, Rush University Medical Center, Chicago, IL 60612, USA;; 2 Institute of Biomechanics, Hamburg University of Technology, Hamburg, Germany; 3 Endolab Mechanical Engineering GmbH, Ahornweg 8, 83083 Riedering, Germany;

**Keywords:** UHMWPE wear, albumin, Total Joint Replacement

## Abstract

Total Joint Replacement (TJR) devices undergo standardized wear testing in mechanical simulators while submerged in a proteinaceous testing solution to mimic the environmental conditions of artificial joints in the human body. Typically, bovine calf serum is used to provide the required protein content. However, due to lot-to-lot variability, an undesirable variance in testing outcome is observed. Based on an earlier finding that yellowish-orange serum color saturation is associated with wear rate, we examined potential sources of this variability, by running a comparative wear test with bilirubin; hemin; and a fatty acid, oleic acid, in the lubricant. All these compounds readily bind to albumin, the most abundant protein in bovine serum. Ultrahigh molecular weight polyethylene (UHMWPE) pins were articulated against CoCrMo discs in a pin-on-disc tribometer, and the UHMWPE wear rates were compared between lubricants. We found that the addition of bilirubin increased wear by 121%, while hemin had a much weaker, insignificant effect. When added at the same molar ratio as bilirubin, the fatty acid tended to reduce wear. Additionally, there was a significant interaction with respect to bilirubin and hemin in that UHMWPE wear rate decreased with increasing fatty acid concentration. We believe the conformational change in albumin by binding bilirubin makes it more likely to form molecular bridges between UHMWPE and the metal counterface, thus increasing adhesive wear. However, fatty acids compete for binding sites on albumin, and can prevent this conformational change. Hence, the protein is stabilized, and the chance for albumin to form bridges is lowered. Ultimately, UHMWPE wear rate is driven by the competitive binding of bilirubin and fatty acid to albumin.

## 1. Introduction

Total Joint Replacement (TJR) is a successful clinical procedure to alleviate pain and restore the function of damaged articular joints in the human body. Most of the replacement bearings use a hard-on-soft combination, with ultrahigh molecular weight polyethylene (UHMWPE) as the soft partner of the bearing couple. In the development of new TJR devices, UHMWPE particle release is of concern. Therefore, prosthetics are subjected to standardized [1–4] simulated wear tests, while samples are submerged in a proteinaceous solution. This solution is intended to replicate the synovial fluid, and contains water, albumin (the most abundant protein in synovial fluid [5]), buffers, and often antibiotics and antifungal agents. Previous studies dealing with metal-on-UHMWPE bearings [6,7] have shown that the protein content in the testing lubricant has a large influence on the polyethylene wear rate. Thus, bovine serum, containing albumin similar to that of human, is mixed in to reach a specific protein concentration as dictated by ISO and ASTM guidelines.

However, even following these guidelines, and controlling for total protein level, polyethylene wear remains variable. As an animal product, bovine serum contains a variety of macromolecules with some degree of lot-to-lot variability [8]. This variability seems reflected in wear testing results. In a recent study [9], different lots from the same stock keeping unit (SKU), purchased from the same supplier, and diluted to the same protein concentration, yielded wear rates that varied as much as 30% from lowest to highest, despite the use of identical TJR devices. This was much larger than the sample-to-sample variation within the same lot (6%). In order to further improve the reliability of standardized wear testing, understanding and addressing this source of variability seems imperative.

It is well documented that different protein fractions in bovine serum, and their state (e.g., natural, agglomerated, cleaved, etc.), influence UHMWPE wear [10]. In addition, literature is available discussing the presence and absence of serum additives on wear [7,11,12]. However, relatively little is known about the wear effect of molecular compounds that occur concomitantly in bovine serum. In her thesis [9], Scholl found that, despite having the same total protein concentration, lighter colored solutions tended to lower, while darker colored solutions tended to higher, wear rates. Hence, statistically speaking, wear rate displayed a moderate to strong dependency on yellowish-orange color saturation of the testing fluid (r = 0.6; p < 0.05). The source of this varying color saturation could be due to the presence of bilirubin, an orange-brownish compound generated in the process of breakdown of red blood cells. Bilirubin readily binds to albumin [13], and while bovine serum is processed to remove all cells from blood, bilirubin may well be present. Similarly, intermediate turnover products from red blood cells, like hemin, a dark green iron-containing porphyrin, can also bind to albumin.

Bovine Serum Albumin (BSA) is a ~66kDa protein that, next to regulating protein-induced osmotic pressure in the bloodstream, also functions as a transport molecule [14]. Its numerous binding sites allow it to interact with cations, smaller proteins, hormones, fatty acids, and many pharmaceuticals [13]. The role of BSA on UHMWPE wear has been studied extensively [15–18]. In our previous work [18], we extended the concept of albumin adhesion by Widmer et al. [19] and proposed that in vivo PE wear is generated by an adhesive binding process, driven by protein interactions with both joint counterfaces. In this model, hydrophilic areas of albumin adsorb onto the metal surface, and the hydrophobic area binds to the polymer surface when it is brought close in contact. This creates molecular bridges that, in the case of a sliding contact, may rupture at the polyethylene surface, thus pulling off UHMWPE particles from the bulk. Our group has shown when the albumin is cleaved, the albumin is less likely to form these bonds and the adhesive wear rate decreases. This suggests that conformational changes to BSA or alterations in its structural integrity by the presence of bound macromolecules may affect the chance of forming metal-to-polyethylene bridges, and in turn may change the wear rate.

While the presence of bound bilirubin might be a source of variability, fatty acids (FA) are also present in BSA and may compete with available bilirubin BSA binding sites [20,21]. In general, lipids in serum have been shown to influence UHMWPE wear rates [22]. The lipid profile of bovine serum can be altered by changes in diet [23] and the time between the collection of blood and feeding. After changes to New Zealand animal welfare regulations took effect in 2018, the allowable time between a calf’s slaughter and its final feed was shortened considerably [24]. Anecdotally, one supplier of Bovine Calf Serum found after the feeding change that their new serum had to be subjected to additional filtering to reduce lipid turbidity, and that the product had to be brought back into their historical range. However, since FAs bind to albumin, filtering may not be effective.

Our primary goal was to evaluate the effect on wear of bilirubin bound to BSA. Based on the observations of Scholl [9], we hypothesized that BSA saturated with bilirubin will cause a higher wear rate than BSA without bilirubin. Further, we were interested if related compounds to bilirubin, like hemin, show similar effects on wear. Lastly, we asked the question if the FA oleic acid, which uses similar binding sites on BSA like bilirubin, could mitigate or even eradicate the effect. In order to address these questions, we conducted two rounds of wear tests where we compared plain BSA, bilirubin/BSA, hemin/BSA, and FA/BSA. Further, we tested the binding interaction of FA with bilirubin- and/or hemin-BSA solutions with the addition of various amounts of FA to the respective lubricants.

## 2. Materials and Methods

### 2.1. Testing Apparatus

**Figure 1.**
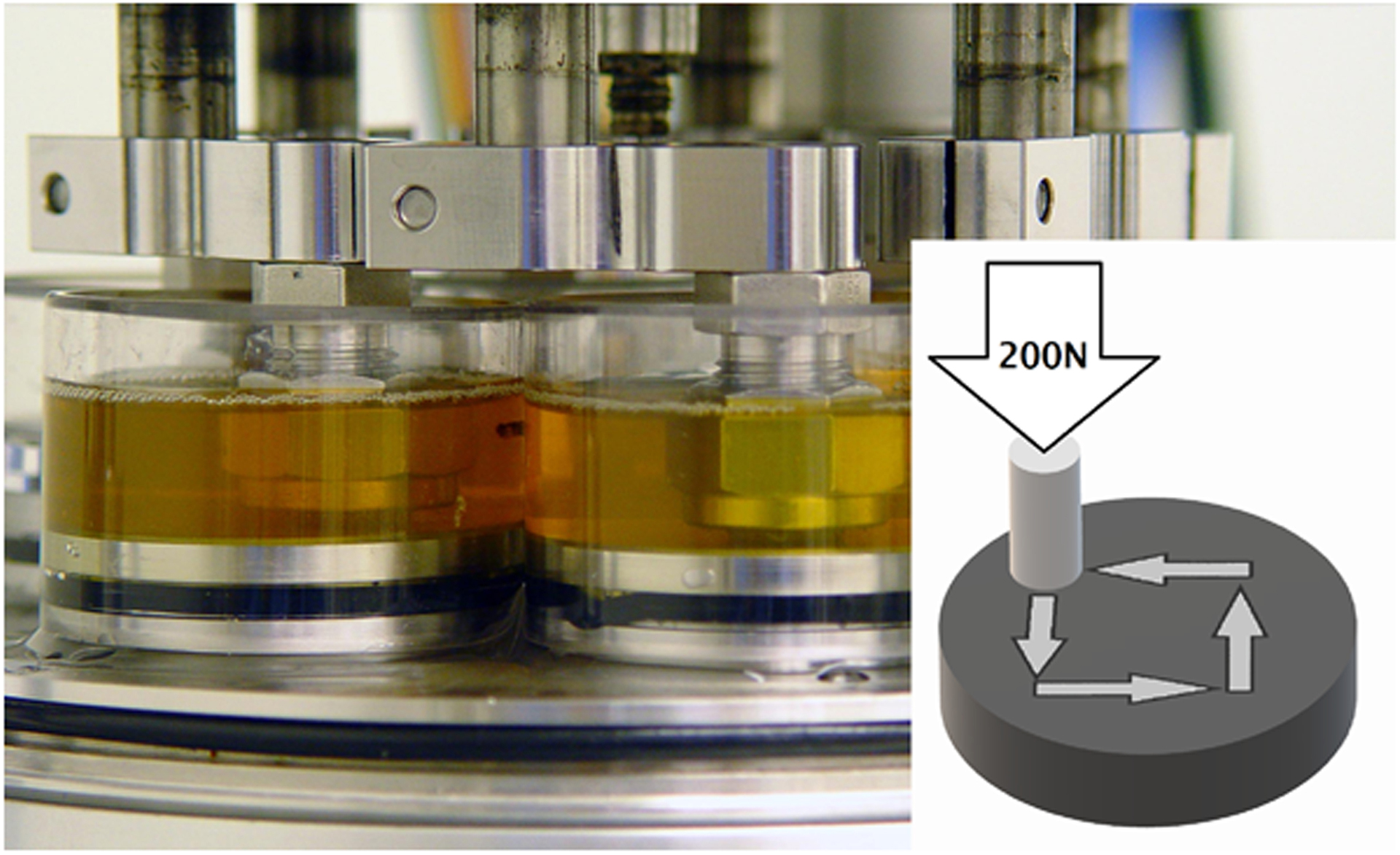
Photograph of the 6-station AMTI OrthoPOD machine with testing solution but without heating bath. Image insert is simplified view of ultrahigh molecular weight polyethylene (UHMWPE) pin on CoCrMo disc configuration. Each pin was loaded to 200 N, and moved in a 15 mm square pattern (gray arrows) while submersed in 15mL of testing solution. The cycle was completed in 1 s.

All tests were conducted on a pin-on-disk (POD) wear testing apparatus (OrthoPod, AMTI, Watertown, MA, USA) seen in Figure 1. This apparatus has six stations, each consisting of a CoCrMo disk articulating against a UHMWPE pin. Pin motion was programmed to execute a 15 × 15 mm square cycle to be completed within one second. This led to perpendicularly crossing motion trajectories on the pin and an average sliding velocity of 60 mm/s, which is similar to that occurring in a hip joint. A load of 200 N was applied to the pin, yielding a technical stress of 2.8 MPa on the UHMWPE pin. Again, this is similar to the average contact stresses of artificial joints. Each of the stations was confined by a Plexiglas cylinder containing 15 mL of lubricant, surrounded by a water bath in excess of 1L, kept at 37 °C.

### 2.2. Counterfaces

Twelve low-carbon CoCrMo discs were sequentially polished, then measured to have an average roughness of Ra = 5.11 ± 50 nm using white light interferometry (Zygo Corp. Middlefield, CT, USA). These disks were originally machined and donated by a major implant manufacturer (Zimmer Inc., Warsaw, IN, USA) for wear testing against polyethylene [18]. Fourteen 9.5 mm diameter GUR 1050 UHMWPE pins (Orthoplastics Ltd., Landcashire, UK) were purchased (Table 1). The pins were gamma-irradiated (30 ± 5 kGy) in nitrogen, then soaked in DI water until mass change stabilized. Before soaking, pins were stored at −80 °C to slow the rate of oxidation. For each round of testing, one pin was used as a soak control. During testing it was stored at 37 °C in DI water—all wear rates presented have been corrected by this weekly soak pin mass change.

### 2.3. Lubricants

For all lubricants, the mass concentration of BSA was mixed with a buffer solution (0.154 M NaCl, 0.223 M Tris, 0.538 mM EDTA, pH 7.6) to match the ISO 14242–1 standard for hip wear testing, 30 g protein/L [2]. Additionally, in all cases, sodium azide (3 g/L) and penicillin/streptomycin (100 IU/mL and 100 μg/mL) were added to prevent bacterial growth. One control station was run with a cold alcohol precipitation fraction V (Sigma # A2153, St. Louis, USA) reference BSA. All other lubricant conditions used a fatty acid and protease free heat shock extracted BSA (Sigma # A7030).

Four lubricants were tested: “plain” BSA, bilirubin-BSA, hemin-BSA, and FA-BSA. For bilirubin lubricants, 73.6 mg (Sigma # B4126) was dissolved in 4 mL of 0.1 M NaOH, then added to a solution of 1.2 g BSA and buffer, then diluted to 40 mL. This equaled 3.15 mM of bilirubin, yielding a 7:1 bilirubin to BSA ratio. For hemin lubricants, 82.2 mg (Sigma catalog #51280) was dissolved in 4 mL of 0.1 M NaOH, then added to a solution of 1.2 g BSA and buffer, then diluted to 40 mL. This equaled 3.15 mM of hemin, yielding a 7:1 hemin to BSA molar ratio. Being the most common monounsaturated fatty acid found in both human and bovine serum, Oleic acid (Sigma # O1008) was selected for the FA-BSA lubricant. To make 40 mL of solution, 40 μl of 3.17 M stock oleic acid was diluted with 360 μl of absolute ethanol, then added to 39.6 mL of already prepared BSA (3% w/v) lubricant. This yielded a final concentration of 3.17 mM, or a 7:1 ratio of FA to BSA.

The interaction of FA on wear of bilirubin- and hemin-BSA solutions was also examined by the addition of two concentrations of FA. For Low FA lubricants, 3.17 mM of FA was used; for High FA lubricants, 6.34 mM of FA was used (or a 14:1 ratio of FA to BSA).

All lubricants were prepared freshly and independently for each testing interval.

### 2.4. Wear Tests

Wear testing was conducted in two rounds up to two million cycles (MC). Each round consisted of four testing intervals of 0.5 MC each. In the first round, the lubricants “plain BSA”, “bilirubin-BSA”, and “FA-BSA” were investigated. In Round 2, FA-BSA was replaced by “hemin-BSA”. For each round, every station was fitted with a new CoCrMo disk and a new UHMWPE pin, thus generating twelve tribosystems. For each round, lubricants were run in duplicate, and stations were randomized, in that there was no station overlap between plain BSA and bilirubin-BSA in Rounds 1 and 2. In order to address the interaction effect of bilirubin and hemin with fatty acids, Round 2 was continued with the addition of high and low amounts of FA for 1 MC each. One of the BSA only stations served as a control station throughout. After every testing interval of 0.5 MC the test was paused, the system was cleaned, the lubricant was replaced, and the weight of the pins was gravimetrically assessed with a high precision balance (0.01 mg resolution, Mettler-Toledo, Columbus, OH, USA) following the ISO 14243–2 [25] measurement protocols. In order to minimize environmental influences on the weight measurements, the latter were performed in a temperature and humidity-controlled glove box. Weight loss was determined by subtracting the measured weight of the pin from the weight that was recorded before the current wear cycle interval started and correcting the delta value with the weight gain of the passive soak pin.

### 2.5. Data Analysis

Since lubricants were freshly prepared for each interval, the wear rates of each interval were used for analysis. These values were multiplied by 2, to express each wear rate in mg/MC. The first 500,000 cycle interval was excluded, to eliminate “run-in” wear. To address the effect of lubricant (BSA, hemin-BSA, bilirubin-BSA, FA-BSA), a mixed model was used to examine whether or not wear rates differed between lubricants. Model inputs consisted of 36 wear rates; lubricant was considered as the fixed effect, while “tribosystem” was considered as the random effect. The concentration of fatty acid and its interaction with wear rate was statistically evaluated using mixed modeling, too. All calculations were performed using the MIXED procedure in SAS software (SAS Institute Inc., Cary, NC, USA). Type 3 sums of squares method was used for estimating the variance components. Bonferroni’s correction was used to correct for Type I error due to multiple comparisons.

## 3. Results

At the conclusion of the first round of testing, CoCrMo disks looked polished, with some minor scratches for all testing solutions. Polyethylene pins had both slightly polished and rough areas, with moderately sized protrusions in the case of BSA. For pins run in Low FA/BSA, the surface was smoother, with more, but smaller protrusions. Pins run in bilirubin/BSA solution appeared highly polished, and contained protrusions exclusively located in the center of the pins.

In all twelve tribosystems, the UHMWPE pins wore at a highly linear rate after running-in (R^2^ > 0.99). There was a significant difference in wear rates between the lubricants, F(3, 24) = 46.72, p < 0.001. The estimated wear rate was 11.27 mg/MC (95%CI [9.21, 13.3]) for BSA, 24.93 mg/MC (95%CI [22.87, 26.99]) for bilirubin/BSA, 15.51 mg/MC (95%CI [12.60, 18.42]) for hemin/BSA, and 7.41 mg/MC (95%CI [4.50, 10.31]) for Low FA/BSA. Previously published [26] wear tests run on the same machine with the same loading and motion profiles, but using a standard calf serum-based solution, had a higher wear rate than Low FA/BSA but a lower rate than pure BSA. This data and the calculated specific wear rates are shown in Table 2. According to the specific wear rates, all pins ran in the mild wear regime. Pairwise comparison with Bonferroni’s correction revealed that bilirubin-BSA was different from the other three luricants (all p < 0.001), and hemin- and Low FA-BSA were different (p = 0.003). Pairwise comparison of BSA and hemin-BSA, and BSA and Low FA-BSA, were found not to be significant (p = 0.130 and 0.209, respectively).

**Figure 4.**
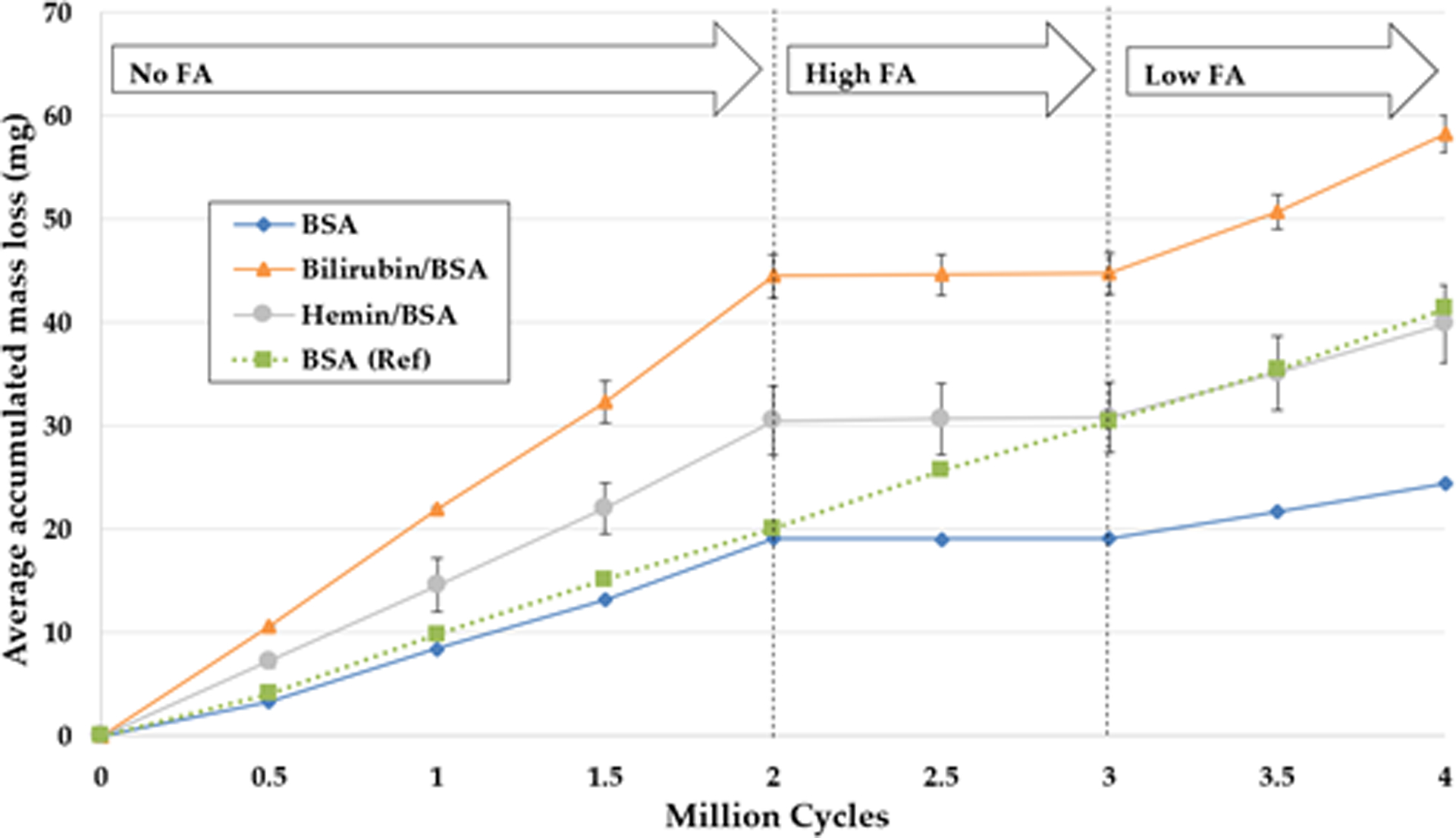
Total accumulated mass loss of six stations throughout the second round of testing. Bilirubin/BSA and hemin/BSA are represented by averages of two stations. Error bars represent the standard deviation. The vertical lines indicate where the FA content of the lubricant was changed. Arrows at the top of the graph indicate the concentration of FA in the lubricants for the time points. One station, BSA (Ref) did not have FA added during the test run.

**Figure 5.**
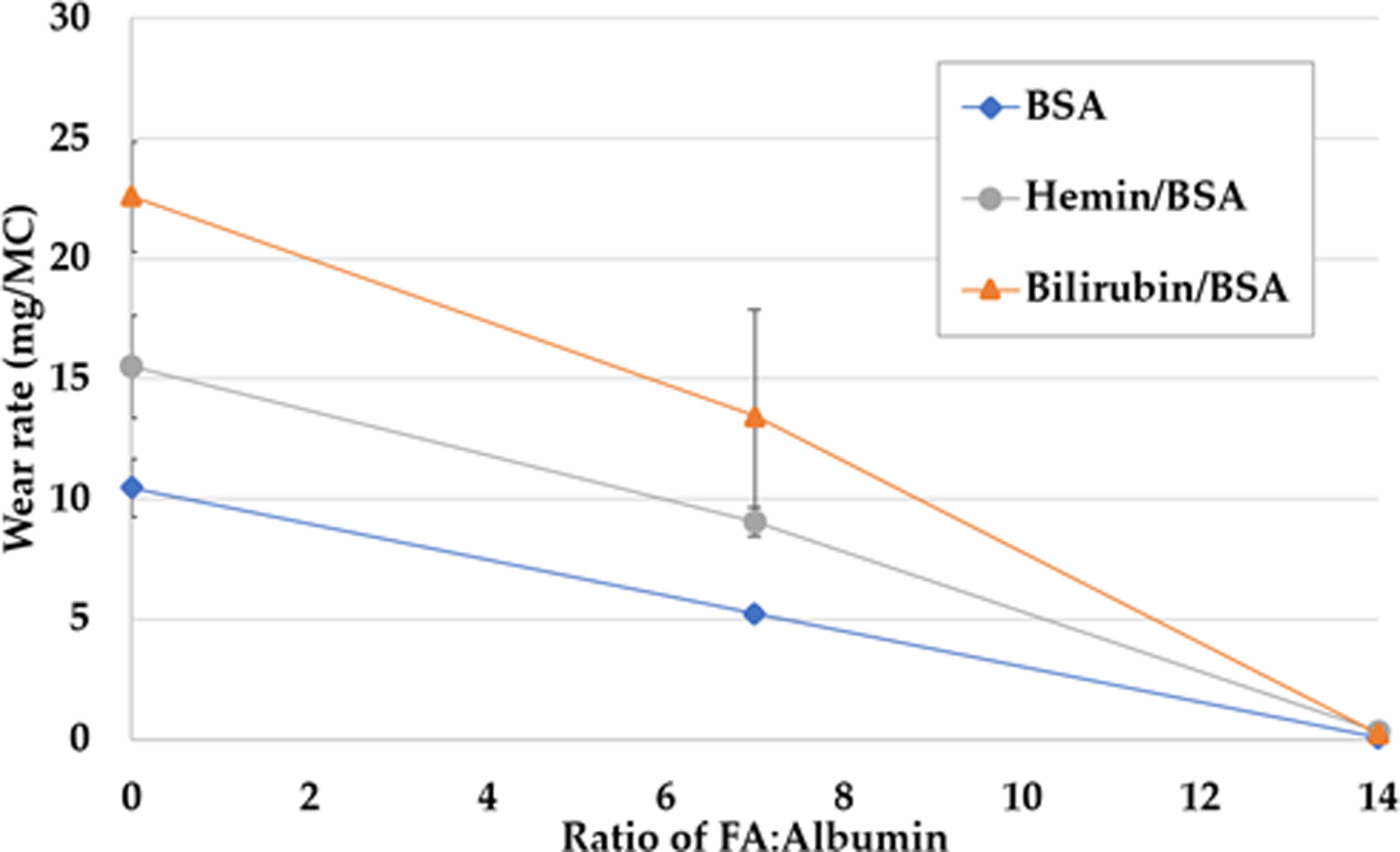
Representation of average wear rate for differing FA concentrations in the second round of testing. Error bars represent standard deviation.

For the continuation experiment in Round 2, the effect of lubricant and concentration of the oleic acid (FA) and their interaction were examined. The BSA control station, which ran consistently over eight measurement intervals, generated a highly linear wear rate of 10.37 mg/MC (R^2^ = 0.9995), suggesting reliable testing and measurement conditions (Figure 4). There was a significant interaction between lubricant and concentration of FA, F(4, 26) = 7.51, p < 0.001. With respect to bilirubin and hemin, as the concentration of fatty acids increased, the wear rate decreased. Remarkably, the addition of High FA caused the linear wear rates to drop 98–99%, to near zero (Figure 4). The one station with BSA was reduced to 0.07 mg/MC, and averages for bilirubin/BSA and hemin/BSA decreased to 0.26 mg/MC and 0.33 mg/MC, respectively. Compared to No FA, Low FA lowered BSA, bilirubin/BSA, and hemin/BSA wear rates by 50%, 41%, and 40%, respectively. This effect can be seen in Figure 5. For hemin and bilirubin, we found these differences to be significant, but for BSA, this was only true when comparing the No and High FA conditions (Table 3).

## 4. Discussion

The impetus for this study was the unexpected result by Scholl [9], finding saturation of serum color associated with UHMWPE wear rates. Color variation in serum lots is often attributed to the presence of hemoglobin, but C.S. found no association between wear rates and serum iron content when recently tested (personal communication). While the exact source of this color difference was not determined in reference [9], an albumin-bound product of the normal degradation of heme seemed to be the most probable reason. This study confirmed the hypothesis that bilirubin bound to BSA increases the wear rate of UHMWPE, actually more than doubling it compared to plain BSA. Additionally, the study showed that macromolecules other than bilirubin can bind to albumin, thereby potentially influencing UHMWPE wear rates. Particularly, common fatty acids like oleic acid tested in these experiments are in competition with bilirubin regarding albumin-binding sites and show the exact opposite effect, namely, cutting the BSA wear rates in half. Thus, the presence of fatty acids may effectively cancel out the wear increasing effects of bilirubin, while excessive amounts of the same may inactivate binding to UHMWPE altogether and reduce wear rates in the range of pure water lubrication.

Bilirubin binds to hydrophobic binding sites in BSA. The binding of bilirubin causes conformational changes in the BSA molecule [27]. These conformational changes likely expose additional hydrophobic amino acids toward the surface of the molecules. The hydrophobic patches on different BSA molecules then lead to the aggregation of the BSA, making adhesion more potent: the aggregated BSA with additional hydrophobic character on their surfaces will bind more avidly to the hydrophobic surface of the PE and thus lead to greater adhesive wear.

In addition, the articulation of the metal disc with the polyethylene pin will cause a mechanical denaturation of BSA over time. Denaturation of BSA leads to more hydrophobic amino acids on its surface, which are normally buried in the center of the native molecule. We speculate that these hydrophobic patches on different BSA molecules cause them to aggregate and bind more avidly to the polyethylene surface, increasing wear. In this case, the additional hydrophobic patches on the denatured BSA and polyethylene are stabilized by the high concentrations of the hydrophobic bilirubin molecule. As BSA molecules aggregate, the large aggregates scatter light and the previously clear solution becomes cloudy. This is exactly what happened during the articulation experiments. After some time in the testing protocol, the initially clear BSA solution became somewhat cloudy. In the course of these experiments, when the bilirubin was added to the solutions, the cloudiness occurred at earlier times than when bilirubin was not present. If a clear BSA solution is treated with increasing concentrations of ammonium sulfate, the BSA will aggregate and the solution will also become cloudy. Although speculative, the mechanism for this salt-mediated aggregation is thought to be due to the ammonium sulfate dehydrating the water solvation layers on the BSA [28] allowing exposed hydrophobic patches on different molecules to aggregate.

Bilirubin and fatty acids have several binding sites [13] on serum albumin. They both have preferred high affinity sites [27], but they also likely share some lower affinity sites. Bilirubin is less polar than oleic acid, so the addition of oleic acid to bilirubin decreased the hydrophobic character of the molecules bound to the BSA. This led to decreased wear. At a 7:1 molar ratio of FA to BSA, the Low FA was slightly below the binding capacity of albumin [29]. At the High FA, 14:1 molar ratio, the excess oleic acid likely formed micelles, which lubricated the metal-polyethylene interface, also limiting the chance of albumin bridging, and reducing wear to very low rates that are typically seen with pure water lubrication [30].

The wear rates and mechanisms of this study agree well with simulator studies. Using POD wear tests with the same load and kinematic input as used here, Mell et al. [26] were able to predict knee simulator wear rates computationally. In addition, the observed wear features (particularly the protrusions) have been observed during hip simulator wear testing, as well as on in vivo components after retrieval [31].

While the relevance of our findings is immediate to simulated implant testing, wear phenomena in synovial fluid may be different. Additionally, some groups seek to make testing solution more “synovial-like” by the inclusion of hyaluronic acid [32] and phospholipids [33]. As these formulations are not currently used in standardized testing, they were not examined. Additionally, while albumin is the main protein constituent of newborn calf serum, gamma globulin, and the ratio of globulin to albumin, have been shown to have significant effects on wear rates [6].

One limitation of this study was that only one fatty acid, oleic acid, and two hemoglobin metabolites were studied that demonstrated varied effects on wear in this tribosystem. Serum also contains many other hydrophobic molecules, including cholesterol, triglycerides, phospholipids and different free fatty acids. Such molecules may have tribological effects similar to or different than those studied, depending on their molecular structure, concentrations, and binding properties with BSA. For example, triglycerides are neutral molecules with three fatty acids in ester linkages to glycerol and therefore might behave like oleic acid, while phospholipids have two fatty acids bound to glycerol plus a charged phosphate and amino groups, which might behave differently due to their charge.

Another limitation of the study was that wear effects were evaluated at pH 7.6 only. In the future, acidic conditions should be tested as well since they are relevant during joint inflammation and might affect interactions between lubricant constituents. Next, for this study we did not investigate lubricant viscosities, which are known to have effect on the lubrication state. We believe, however, that these values are reasonably close to previously published data of 50% diluted bovine calf serum [34]. Last, our soak control pins were kept in DI water and, thus, not exposed to proteins and lipids that might affect the weight gain. While we have unpublished lab data that document that polyethylene pins gain a similar amount of weight in both diluted bovine serum and DI water, we do not have these data for lipids. However, even under the assumption of 100% absorption in the case of high FA solution, this would only account for 0.026 mg of weight gain. Hence, most of the determined wear rates stay rather unaffected.

With improved implant materials and design, the wear rates of TJR devices are expected to further decrease. This makes it important to increase resolution and accuracy of preclinical wear testing. Due to the low magnitude of wear rates, all unaddressed sources of testing variability make it increasingly difficult to compare results between experiments, and continue to evaluate new designs. The biochemical origin of the variability in bovine serum needs to be further elucidated, and potentially controlled for. Serum lot variability is a well-known issue in biology [35], and serum suppliers will often provide lot-specific results from osmolarity, protein, pH, and sterility assays to customers. However, these certificates of analysis do not provide bilirubin and fatty acid levels bound to albumin. We hope this work raises awareness of this unaddressed problem in the orthopedic testing community.

## 5. Conclusions

We have demonstrated albumin binding macromolecules can significantly alter UHMWPE wear rates. The addition of bilirubin to serum albumin was found to increase wear rates. Conversely, the addition of FA can reduce or almost eliminate adhesive UHMWPE wear depending on concentration. Further studies are needed to quantify bound macromolecules in commercial serum lots, and to correlate these numbers with wear rates. The presence of these macromolecules should be an area of concern for all wear labs desiring lower inter-test variability. A definition of an acceptable range for these molecules may be required, similar to the current standardization of protein levels in testing solutions.

## Figures and Tables

**Figure 2.**
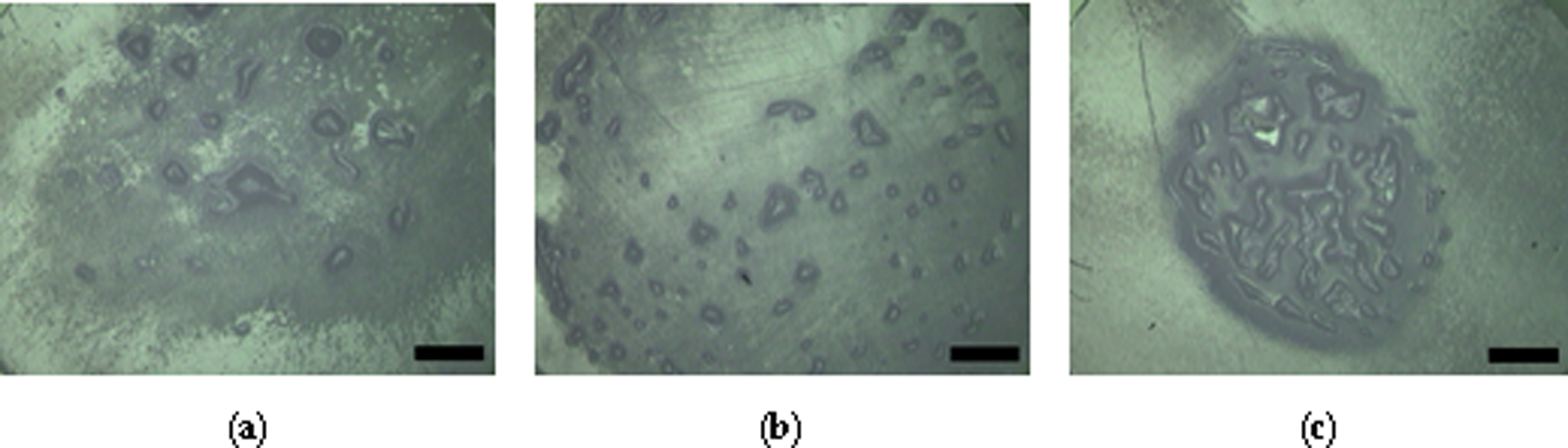
Representative micrographs of the center of pins after 2 MC wear cycles. The pins were run in (**a**) BSA, (**b**) Low FA/BSA, and (**c**) bilirubin/BSA testing solutions. Scale bar is 1 mm.

**Figure 3.**
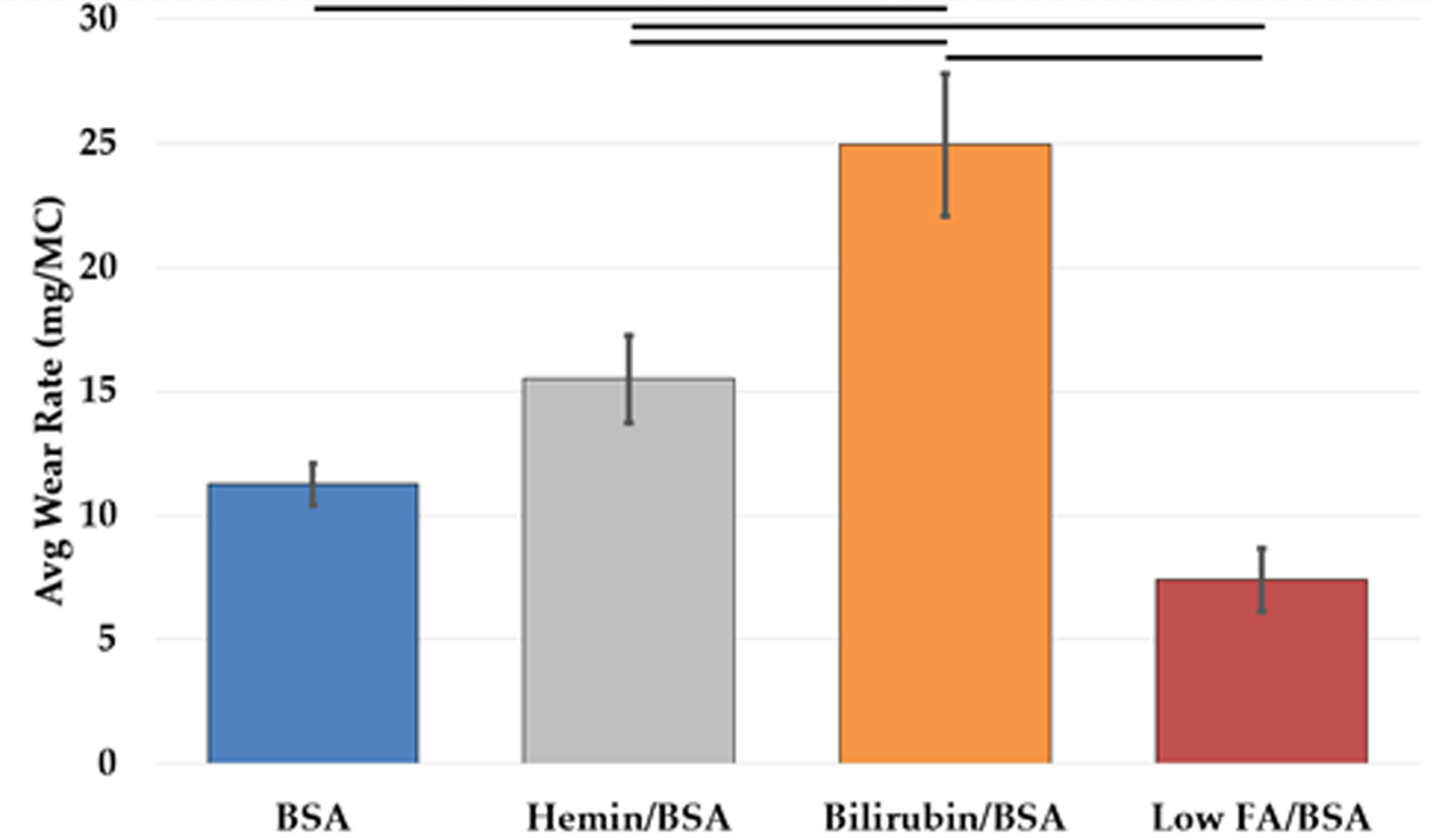
Comparison of average wear rates for all lubricant conditions across both testing rounds. Horizontal bars indicated pairwise adjusted p < 0.01. Error bars indicate standard deviation.

**Table 1.** Material properties for compression-molded GUR 1050 medical grade UHMWPE.

Property	ASTM Standard	Test Result	Units
Density	D1505–10	931	kg/m^3^
Tensile Stress at Yield	F648–14 (D638–10)	22.1	MPa
Ultimate Tensile Strength	F648–14 (D638–10)	63	MPa
Elongation at Break	F648–14 (D638–10)	420	%
Izod Impact Strength	F648–14 (D256)	102.5	kJ/m^2^

**Table 2.** Model fitted estimated of wear rate and specific wear rate.

Lubricant	Wear Rate (mg/MC)	Specific Wear Rate (mm^3^/Nm)
BSA	11.27	9.40E-7
Hemin/BSA	15.51	1.36E-6
Bilirubin/BSA	24.93	2.12E-6
Low FA/BSA	7.41	5.96E-7
Newborn Calf Serum Solution [26]	8.41	7.53E-7

**Table 3.** Comparison of estimated wear rates and Bonferroni adjusted p-values in the second round of testing with varying concentrations of FA addition.

Lubricant	FA	FA	Estimate	SE	Adj P	Adj Lower	Adj Upper
BSA	High	Low	−5.17	2.07	0.6942	−12.59	2.25
	High	No	−10.4	1.89	0.00003	−17.17	−3.63
	Low	No	−5.23	1.89	0.373	−12	1.54
Bilirubin	High	Low	−13.19	1.47	<0.0001	−18.43	−7.94
	High	No	−22.33	1.34	<0.0001	−27.11	−17.54
	Low	No	−9.14	1.34	<0.0001	−13.92	−4.35
Hemin	High	Low	−8.71	1.47	0.0001	−13.96	−3.47
	High	No	−15.17	1.34	<0.0001	−19.96	−10.39
	Low	No	−6.46	1.34	0.0019	−11.25	−1.67
